# No evidence for tactile entrainment of attention

**DOI:** 10.3389/fpsyg.2023.1168428

**Published:** 2023-05-26

**Authors:** Ulrich Pomper

**Affiliations:** Department of Cognition, Emotion, and Methods in Psychology, Faculty of Psychology, University of Vienna, Vienna, Austria

**Keywords:** entrainment, tactile, somatosensory, alpha, rhythm, oscillation, attention

## Abstract

Temporal patterns in our environment provide a rich source of information, to which endogenous neural processes linked to perception and attention can synchronize. This phenomenon, known as entrainment, has so far been studied predominately in the visual and auditory domains. It is currently unknown whether sensory phase-entrainment generalizes to the tactile modality, e.g., for the perception of surface patterns or when reading braille. Here, we address this open question via a behavioral experiment with preregistered experimental and analysis protocols. Twenty healthy participants were presented, on each trial, with 2 s of either rhythmic or arrhythmic 10 Hz tactile stimuli. Their task was to detect a subsequent tactile target either in-phase or out-of-phase with the rhythmic entrainment. Contrary to our hypothesis, we observed no evidence for sensory entrainment in response times, sensitivity or response bias. In line with several other recently reported null findings, our data suggest that behaviorally relevant sensory phase-entrainment might require very specific stimulus parameters, and may not generalize to the tactile domain.

## 1. Introduction

Sensory information from our environment features an abundance of temporal regularities, such as in animal movement patterns, vocal communication, or exploratory behaviors like saccading or whisking (Schroeder et al., 2010; Ding et al., 2017; Kotz et al., 2018). These temporal regularities are mirrored by rhythmic oscillatory activity patterns in the brain, which reflect ongoing changes in neural excitability and thus the capacity to process information (Buzsáki, 2006; Wang, 2010). In a broad definition, the term ‘entrainment’ denotes the idea that internal neural oscillations can synchronize with external regular sensory patterns, aligning moments of increased processing capacity with incoming task relevant information, and thus optimizing behavior (Haegens and Zion Golumbic, 2018; Rimmele et al., 2018; Lakatos et al., 2019; Obleser and Kayser, 2019).

Past research has observed evidence for entrainment in both purely behavioral (Lawrance et al., 2014; Spaak et al., 2014; Hickok et al., 2015) and electrophysiological studies (Mathewson et al., 2012; de Graaf et al., 2013; Spaak et al., 2014; Bauer et al., 2021). That is, they found that following rhythmic sensory stimulation at a given frequency *f*, behavioral performance accuracy (e.g., Lawrance et al., 2014; Hickok et al., 2015; Zoefel and Vanrullen, 2015; Bauer et al., 2021) and/ or response times (e.g., Miller et al., 2013; Barnhart et al., 2018) are rhythmically modulated over time at the same frequency *f*. In other words, performance differed between time-points in-phase and out-of-phase with the rhythmic sensory stimulation. Likewise, other studies reported that oscillatory neural activity measured via electro- or magnetoencephalography (EEG or MEG, respectively) is phase-aligned with the rhythmic sensory input (e.g., Mathewson et al., 2012; Bauer et al., 2021), or increased in power at the stimulation frequency *f* during and even after offset of the stimulus (e.g., Spaak et al., 2014). For instance, Spaak et al. (2014), presented their participants with 1.5 s of 10 Hz rhythmic flashes to one visual hemifield and arrhythmic flashes to the other, while recording EEG. Following the offset of the stimulus, they observed an increase in both oscillatory alpha (8–12 Hz) power and intertrial-phase coherence, contralateral to the rhythmic stimulation. Importantly, this effect was accompanied by a cyclic modulation of behavior, with increased detection rates of near threshold visual targets presented out-of-phase compared to in-phase with the preceding flashes. Although several studies have observed such effects not only during but also following the offset of the rhythmic sensory stimulus, the effect appears to diminish quickly within a few 100 ms (e.g., Hickok et al., 2015).

While sensory entrainment has previously been demonstrated in the visual (Mathewson et al., 2012; de Graaf et al., 2013; Spaak et al., 2014) and auditory modalities (Henry et al., 2014; Lawrance et al., 2014; Hickok et al., 2015; Zoefel and VanRullen, 2015), it is currently unknown whether tactile stimuli delivered in a rhythmic fashion can likewise lead to neural entrainment and concomitant rhythmic fluctuations in behavioral performance. Importantly, such a mechanism could aid somatosensory detection and discrimination, for instance when moving one’s fingers across an object to determine structure and composition of the surface material, or for reading braille.

A limited number of studies have so far provided partial evidence in support of behaviorally relevant sensory entrainment in the tactile domain (Ruzzoli and Soto-Faraco, 2014; Gundlach et al., 2016; Fabbrini et al., 2022; Houlgreave et al., 2022). Houlgreave et al. (2022) demonstrated that 12 Hz stimulation of the median nerve causes frequency specific entrainment of contralateral MEG activity, but did not test for potential resulting behavioral effects. Gundlach et al. (2016) reported that transcranial alternate current stimulation at the participants’ individual alpha (8–13 Hz) frequency causes a subsequent phase-entrainment of tactile detectability. Likewise, Ruzzoli and Soto-Faraco (2014) used transcranial magnetic stimulation at 10 Hz over posterior parietal cortex, and observed an overall enhancement of tactile perception, but crucially no cyclic fluctuations in performance indicative of phase-entrainment. This result indicates, that stimulation in the alpha-band sometimes leads to changes not in the phase of ongoing oscillatory activity, but to its power, causing temporally less specific effects on attention and sensory processing.

Together, while these data suggest that direct non-invasive rhythmic stimulation of somatosensory cortex can cause behaviorally relevant entrainment, it is unclear whether such effects generalize to conventional rhythmic tactile sensory stimuli common in everyday life. Moreover, a number of recent studies have failed to observe evidence for entrainment even in the visual and auditory domain, despite using protocols very similar to previous successful demonstrations (de Graaf and Duecker, 2021; Lin et al., 2021; Keitel et al., 2022; Pomper et al., 2022). This additionally questions the ubiquity of the phenomenon and calls for further research into the necessary stimulus parameters.

In the present study, we addressed these issues via a tactile entrainment^1^ experiment, whose stimulation- and analysis procedures were preregistered at the OSF prior to data collection (Pomper et al., 2022), and subsequently carried out accordingly. On every trial, participants were first presented with 2,000 ms of either rhythmic or arrhythmic tactile stimuli at a rate of 10 Hz. Subsequently, tactile targets were presented at detection threshold at several time-points either in-phase or out-of phase with respect to the rhythmic entrainment stimulus. In line with the literature reviewed above, our main hypothesis (1) was that behavioral performance would differ between in-phase and out-of-phase targets following the rhythmic, but not the arhythmic entrainment (*cf.* Spaak et al., 2014; Hickok et al., 2015; Gundlach et al., 2016). Additionally, we also expected that (2) behavioral performance would be overall facilitated following rhythmic compared to arhythmic 10 Hz entrainment (*cf.* Ruzzoli and Soto-Faraco, 2014), and that (3) presumed effects of entrainment would diminish over time following the entrainment offset (*cf.* Hickok et al., 2015). Contrary to our expectations, we observed no evidence of tactile phase-entrainment, and only weak support for the other two hypotheses.

## 2. Methods

### 2.1. Participants

Twenty psychology students (12 female, *M_age_* = 23.9 years, *SD_age_* = 1.6 years) from the University of Vienna participated in the experiment in exchange for course credit. The present sample size was set based on previous studies on sensory entrainment, many of which have included between 20 and 30 participants (Miller et al., 2013; Lawrance et al., 2014; Spaak et al., 2014; Bauer et al., 2021; de Graaf and Duecker, 2021; Sun et al., 2021). Participants reported no history of neurological or psychiatric diseases. Participants further provided written consent prior to the experiment and were treated according to the Declaration of Helsinki.

### 2.2. Apparatus

We conducted the experiment in a dimly lit room. Visual stimuli were presented on a 24.5” G2590PX AOC Gaming LCD monitor (visible part of the display: 54.4 cm x 30.3 cm) with a resolution of 1,920 × 1,080 pixels and a refresh rate of 60 Hz. A constant viewing distance of 57 cm was ensured by a chin rest. The experiment was programmed with and executed via Open Sesame Vers. 3.1. (Mathôt et al., 2012). For the tactile stimuli, a Tactor vibrational unit (Dancer Design, United Kingdom) controlled via the computer’s sound card was used. The stimulus timing was accurate in the range of ± 3 ms, as verified via an oscilloscope connected to the computers sound output. Participants placed their left index finger on the Tactor unit, and their right index finger on the space bar of a conventional “QWERT” keyboard. To mask potential acoustic noise from the tactile stimulator, participants wore earmuffs throughout the experiment.

### 2.3. Stimuli and procedure

**Figure 1:**
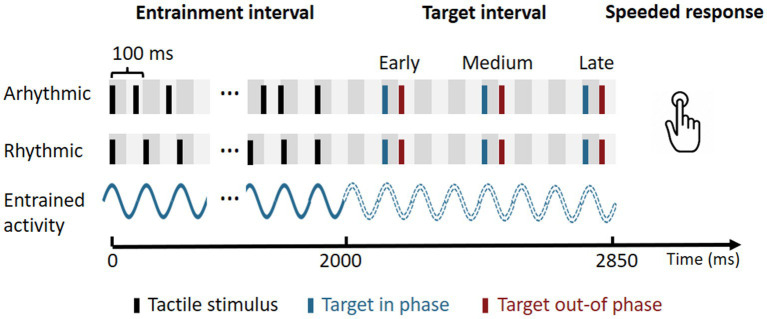
Trial design. Each trial started with an entrainment interval of 2,000 ms duration, during which rhythmic or arrhythmic tactile impulses were presented at a rate of 10 Hz (100 ms cycle length). In the following target interval, a tactile target was presented in 50% of all trials. The target could appear either in-phase or out-of-phase with respect to the rhythmic entrainment stimulation, and during three different time-windows (early, medium, or late). This resulted in a total of six possible target presentation times of 100, 150, 400, 450, 700, and 750 ms following the offset of the entrainment. Participants had to provide a speedy response if they perceived a target. Subsequently, visual feedback on the performance was provided (not shown here). Trials were separated by an inter-trial-interval (ITI) of 800 to 1,200 ms. The continuous blue waveform illustrates the presumed entrained neural activity. The dotted waveform illustrates the expected course of the neural activity, if the stimulus had been continued.

Each trial (Figure 1) started with a 2,000 ms entrainment interval, during which tactile impulses (10 ms duration) were presented. In line with a number of previous studies, we opted for a stimulation frequency in the alpha-band range (Ruzzoli and Soto-Faraco, 2014; Gundlach et al., 2016; Fabbrini et al., 2022; Houlgreave et al., 2022). In the rhythmic condition, impulses were presented regularly at 10 Hz (i.e., separated by a 90 ms interval), resulting in 20 impulses. In the arhythmic condition, likewise, 20 impulses were presented, however separated by randomly varying intervals (from 20 to 200 ms). Importantly, the time-points of the first and last impulse, respectively, were identical in the rhythmic and arhythmic condition. Following the entrainment interval, a tactile target was presented in 50% of trials. The target could appear either in-phase or out-of-phase in relation to the rhythmic entrainment stimulation. Note that, in order to realize a full-factorial design, we also included an in-phase and an out-of phase condition for the arhythmic condition. These labels are not *per-se* meaningful in the arhythmic condition, as there was no entrainment rhythm to begin with. However, both in the rhythmic and in the arhythmic conditions, the in-phase and out-of phase targets were presented at the exact same time following the offset of the final entrainment stimulus. As such, they are comparable with regards to potential general expectancy or foreperiod effects. Further, targets could appear in three different time intervals (early, medium, or late), allowing us to study the duration of potential entrainment effects. This resulted in a total of six possible target time-points: 100 and 150 ms (early in- and out-of phase), 400 and 450 ms (medium in- and out-of phase), and 700 and 750 ms (late in- and out-of phase) in relation to the end of the entrainment stimulus.

The participants’ task was to provide a speeded response with the right index finger if they perceive a target stimulus. Responses had to be given within 1 s following the target. Subsequently, visual feedback on the performance was provided for 200 ms (“correct”; “incorrect”). Trials were separated by a random inter-trial-interval (ITI) of 800 to 1,200 ms.

Independent variables of interest were the preceding Entrainment (rhythmic, arhythmic), as well as the Phase (in-phase, out-of-phase) and the Time (early, medium, late) of the target. Dependent variables were response times (RTs), sensitivity (d’) and response bias (C). The experiment consisted of a total of 720 trials, distributed evenly across the factors Entrainment, Phase, and Time, in a full-factorial design. This resulted in 60 trials per condition, 30 of which contained a target and 30 without a target. The order of trials was pseudo-randomized across participants. Opportunities for self-paced breaks were given after every 72 trials, resulting in 10 experimental blocks. After each block, participants received additional written feedback on their mean RT, hit rate, and false alarm rate in the previous block. Prior to the experiment, individual thresholds for the perception of the tactile target stimuli were assessed through an up-and-down staircase procedure. Stimulus intensities were then continuously adapted throughout the experiment (after every fourth trial) to yield an approximately 75% hit rate. Importantly, intensities were adjusted not separately but across all conditions. This is crucial, since we were interested in performance differences between conditions. Thus, while overall performance was titrated to 75% accuracy and target intensities were the same across conditions, performance was manipulated via the individual conditions of interest (Entrainment, Phase, Time). Additionally, participants completed 15 practice trials before starting the main experiment, to familiarize themselves with the task. The experimental instructions stressed both speed and accuracy of the responses, as well as the necessity to minimize the false-alarm rate.

### 2.4. Data analysis

We only included RTs from trials with correct responses into our analyses and excluded trials with RTs more than 2.5 *SD* above or below the condition’s mean RTs. On average, 1.1% of trials were removed (mean = 7.8 trials, SD = 3.3). For all three of our dependent variables (RTs, d’ and C), we conducted a 2 × 2 × 3 repeated-measures analysis of variance (ANOVA), with the independent variables Entrainment (rhythmic, arhythmic), Phase (in-phase, out-of-phase) and Time (early, medium, late). Significance levels were set to α = 0.05. Significant interactions were followed up by *t*-tests, with reported *p*-values Bonferroni corrected for multiple comparisons. To further investigate the practical relevance the resulting presence or absence of statistical effects, we additionally performed equivalence tests (Lakens et al., 2018) for all follow-up comparisons, which test whether an observed effect falls within or outside a set boundary of relevant size. In line with previous reports of sensory phase entrainment (e.g., Spaak et al., 2014; Hickok et al., 2015; Bauer et al., 2021), this was set to a medium effect size (Cohen’s d = 0.5).

## 3. Results

**Figure 2:**
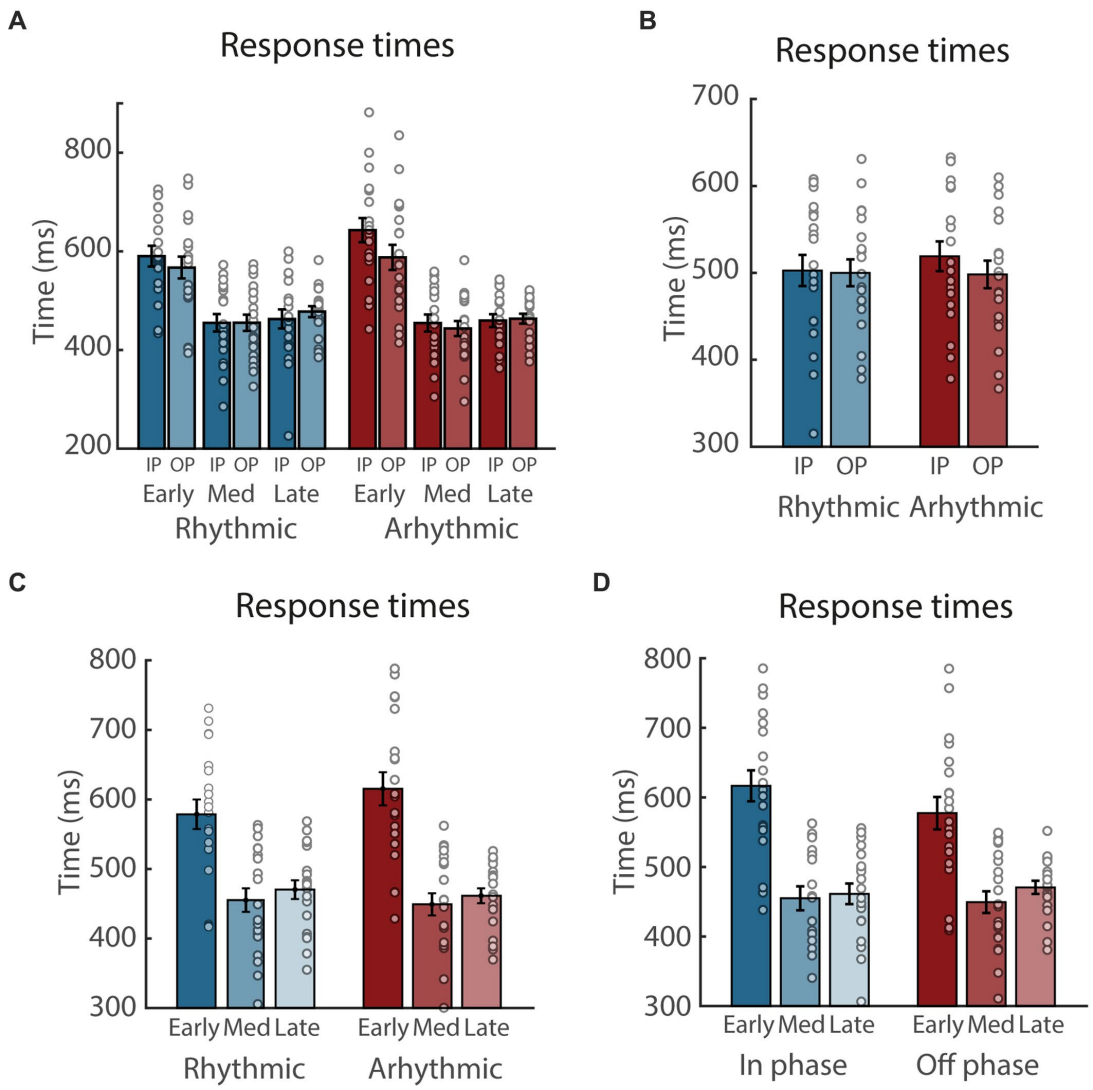
Response time (RT) results. **(A)** RTs, separately for rhythmic and arhythmic trials, as well as targets presented at early, medium (Med), and late time points, and in-phase (IP) and out-of phase (OP) with regard to the rhythmic entrainment. Circles represent individual participants, error bars show standard error of the mean. **(B–D)**: Same as **(A)**, collapsed across the factor Time **(B)**, Entrainment **(C)**, and Phase **(D)**, respectively.

Figure 2A illustrates the overall RT results. The ANOVA yielded significant main effects of Phase (*F*_(1, 19)_ = 4.52, *p* < 0.047, $\eta_{p}^{2}$ = 0.13) and Time (*F*_(1, 38)_ = 116.06, *p* < 0.001, $\eta_{p}^{2}$ = 0.86), as well as interactions between Entrainment and Phase (*F*_(1, 19)_ = 7.81, *p* = 0.012, $\eta_{p}^{2}$ = 0.29), Entrainment and Time (*F*_(1, 38)_ = 13.43, *p* < 0.001, $\eta_{p}^{2}$ = 0.41), and Phase and Time (*F*_(1, 38)_ = 10.80, *p* < 0.001, $\eta_{p}^{2}$ = 0.36). For all other effects, *p*s were > 0.11. To further disentangle the significant interactions, we performed three sets of follow-up pairwise t-tests, each after collapsing the data over one of the factors.

For the present main hypothesis, the crucial interaction is that of Entrainment and Phase, with potentially larger differences between in-phase and out-of-phase in the rhythmic compared to the arhythmic condition, indicative of rhythmic phase entrainment. Surprisingly, however, when exploring the Entrainment x Phase interaction (Figure 2B), we observed significantly slower RTs for in-phase compared to out-of phase targets in the arrhythmic condition (*t*_(19)_ = 3.88, *p* = 0.002, 95% CI [9.6, 32.2], $d_{z}$ = 0.87), but not in the rhythmic condition (*p* = 0.68).

When exploring the Entrainment x Time interaction (Figure 2C), RTs were faster in the rhythmic compared to the arhythmic condition at the early time point (*t*_(19)_ = −4.31, *p* = 0.001, 95% CI [−54.5, −18.9], $d_{z}$ = −0.96) but not at the medium and late time points (both *p*s = 1), providing partial support for the hypothesis of better overall performance following rhythmic entrainment. Additionally, we observed significantly slower RTs in the early compared to the medium and late time windows, both in the rhythmic condition (*t*_(19)_ = 12.60, *p* < 0.001, 95% CI [103.0, −143.0], $d_{z}$ = 2.82; *t*_(19)_ = 7.64, *p* < 0.001, 95% CI [78.7, 138.0], $d_{z}$ = 1.71) and in the arhythmic condition (*t*_(19)_ = 14.89, *p* < 0.001, 95% CI [142.9, 189.6], $d_{z}$ = 3.33; *t*_(19)_ = 9.87, *p* < 0.001, 95% CI [121.2, 186.5], $d_{z}$ = 2.21). No differences were observed between the medium and late time windows (both *p*s > 0.49).

A similar pattern was present when exploring the Phase x Time interaction (Figure 2D), with significantly slower RTs in the early compared to the medium and late time windows, both in the in-phase condition (*t*_(19)_ = 14.43, *p* < 0.001, 95% CI [138.3, 185.2], $d_{z}$ = 3.23; *t*_(19)_ = 11.58, *p* < 0.001, 95% CI [127.3, 183.5], $d_{z}$ = 2.59) and in the out-of phase condition (*t*_(19)_ = 11.86, *p* < 0.001, 95% CI [105.4, 150.6], $d_{z}$ = 2.65; *t*_(19)_ = 6.51, *p* < 0.001, 95% CI [72.5, 141.1], $d_{z}$ = 1.46). Again, no differences were observed between the medium and late time windows (both *p*s > 0.26).

Together, this is indicative of a prevalent main effect of Time, driven by much slower responses at the early compared to later time points.

**Figure 3:**
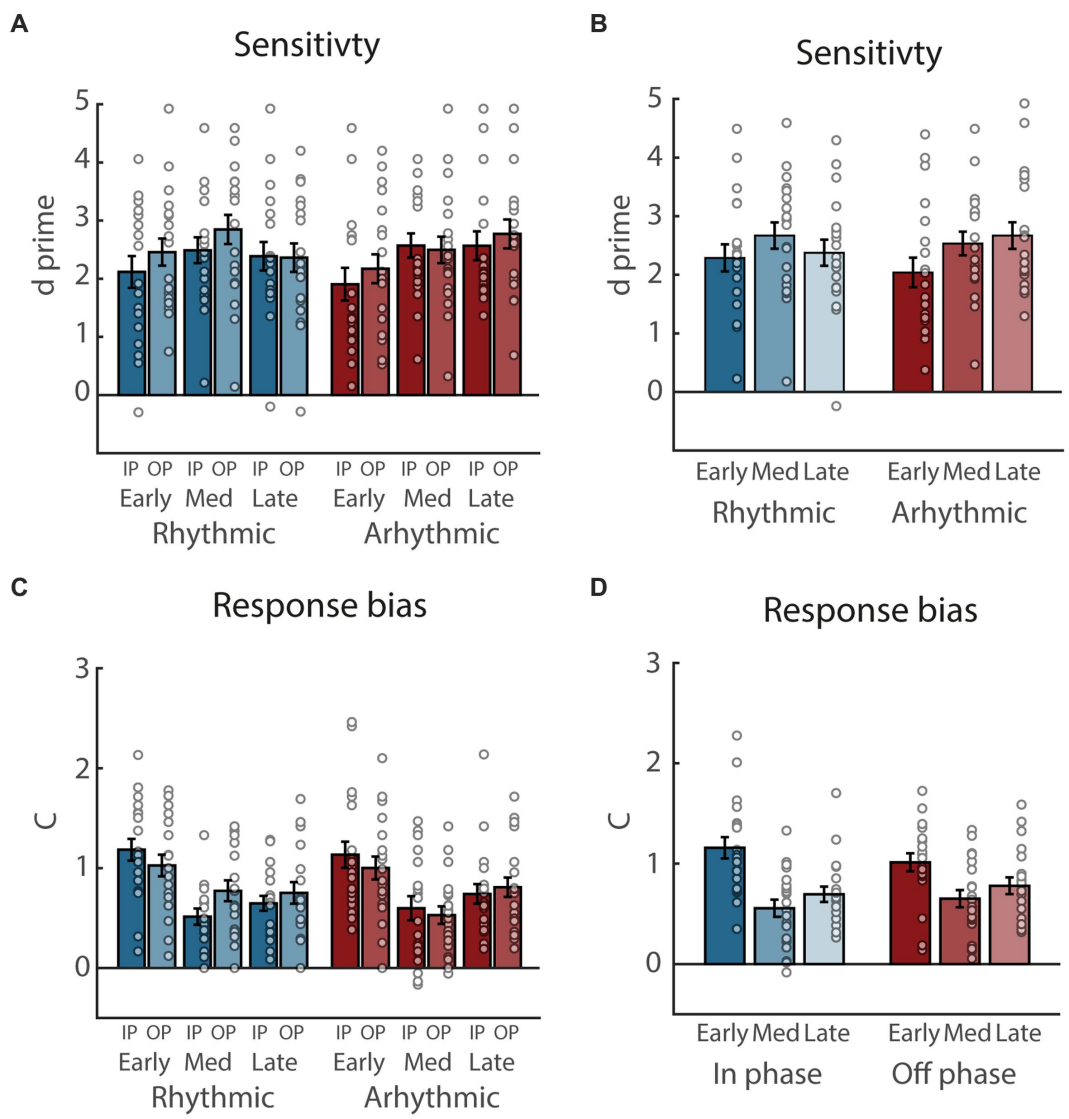
Sensitivity and response bias results. **(A)** Sensitivity, separately for rhythmic and arhythmic trials, as well as targets presented at early, medium (Med), and late time points, and in-phase (IP) and out-of phase (OP) with regard to the rhythmic entrainment. Circles represent individual participants, error bars show standard error of the mean. **(B)** Same as **(A)**, collapsed across the factor Phase. **(C)** Same as **(A)**, for response bias. **(D)** Same as **(C)**, collapsed across the factor Entrainment.

For sensitivity (Figure 3A), the ANOVA yielded significant main effects of Phase (*F*_(1, 19)_ = 4.6, *p* = 0.045, $\eta_{p}^{2}$ = 0.20) and Time (*F*_(1, 38)_ = 7.03, *p* = 0.025, $\eta_{p}^{2}$ = 0.27), as well as an interaction between Entrainment and Time (*F*_(1, 38)_ = 3.50, *p* = 0.040, $\eta_{p}^{2}$ = 0.16). For all other effects, including the crucial Entrainment by Phase interaction, *p*s were > 0.23 ( $\eta_{p}^{2}$ < 0.02). When exploring the Entrainment x Time interaction (Figure 3B), we observed significantly higher sensitivity only in the early compared to the late time window of the arhythmic condition (*t*_(19)_ = 4.0, *p* < 0.001, 95% CI [0.30, 0.96], $d_{z}$ = 0.89). All other *p*s were > 0.055.

Finally, for response bias (Figure 3C), the ANOVA yielded a significant main effect of Time (*F*_(1, 38)_ = 13.89, *p* < 0.001, $\eta_{p}^{2}$ = 0.42), as well as an interaction between Phase and Time (*F*_(1, 38)_ = 5.66, *p* = 0.007, $\eta_{p}^{2}$ = 0.23). For all other effects, *p*s were > 0.21 ( $\eta_{p}^{2}$ = 0.08). When exploring the Phase x Time interaction (Figure 3D), response bias was larger at early compared to medium and late time points in the in-phase condition (*t*_(19)_ = 6.14, *p* < 0.001, 95% CI [0.40, 0.80], $d_{z}$ = 1.37; *t*_(19)_ = 3.52, *p* = 0.014, 95% CI [0.19, 0.74], $d_{z}$ = 0.79), and at early compared to medium time points in the out-of-phase condition (*F*_(1, 38)_ = 4.29, *p* < 0.002, 95% CI [0.19, 0.54], $d_{z}$ = 0.96). All other *p*s were > 0.35.

To further investigate the practical relevance the resulting presence or absence of statistical effects, we additionally performed equivalence tests for all follow-up comparisons reported above (see Table 1 for details). The most important follow-up comparison for our main hypothesis was that between in-phase and out-of-phase RTs in the rhythmic condition, as only the rhythmic condition should induce entrainment leading to subsequent fluctuations in performance. Notably, the equivalence test for this comparison yielded a significant result, suggesting that the observed difference was within the equivalence bounds and not practically relevant. For all other follow-up comparisons, however, the equivalence tests yielded non-significant results, suggesting that the observed effects were outside the equivalence bound and thus relevant. In summary, this further corroborates our original conclusion that the present protocol did not induce tactile phase entrainment effects, while also suggesting that performance between the early, medium, and late time points are more practically relevant then initially assumed.

**Table 1:** Equivalence test results.

Response times											
**Rhy IP vs. OP**	**Early Rhy vs. Arhy**	**Rhy Early vs. Med**	**Arhy Early vs. Med**	**IP Early vs. Med**	**OP Early vs. Med**
*t*	1.87	*t*	2.08	*t*	−10.36	*t*	−12.66	*t*	−12.20	*t*	−9.63
*p*	0.038	*p*	0.974 *	*p*	1 *	*p*	1 *	*p*	1 *	*p*	1 *
**Arhy IP vs. OP**		**Med Rhy vs. Arhy**		**Rhy Early vs. Late**		**Arhy Early vs. Late**		**IP Early vs. Late**		**OP Early vs. Late**	
*t*	−1.64	*t*	0.98	*t*	−5.41	*t*	−7,63	*t*	−9.35	*t*	−4.28
*p*	0.941 *	*p*	0.169 *	*p*	1 *	*p*	1 *	*p*	1 *	*p*	1 *
		**Late Rhy vs. Arhy**		**Rhy Med vs. Late**		**Arhy Med vs. Late**		**IP Med vs. Late**		**OP Med vs. Late**	
		*t*	1.07	*t*	−0.40	*t*	−0.79	*t*	−1,3	*t*	−0.08
		*p*	0.148 *	*p*	0.348 *	*p*	0.219 *	*p*	0.105 *	*p*	0.469 *

Sensitivity				Bias			
**Rhy Early vs. Med**		**Arhy Early vs. Med**		**IP Early vs. Med**		**OP Early vs. Med**	
*t*	−0.05	*t*	0.66	*t*	−3.90	*t*	−2.05
*p*	0.479 *	*p*	0.742 *	*p*	1 *	*p*	0.973 *
**Rhy Early vs. Late**		**Arhy Early vs. Late**		**IP Early vs. Late**		**OP Early vs. Late**	
*t*	−1.68	*t*	1.82	*t*	−1.29	*t*	0.22
*p*	0.054 *	*p*	0.96 *	*p*	0.893 *	*p*	0.415 *
**Rhy Med vs. Late**		**Arhy Med vs. Late**		**IP Med vs. Late**		**OP Med vs. Late**	
*t*	0.11	*t*	−1.6	*t*	−0.66	*t*	−0.81
*p*	0.456*	*p*	0.065 *	*p*	0.258*	*p*	0.214*

Results of equivalence tests for all performed *t*-tests, testing for the presence of medium sized effects (Cohen’s *d* = 0.5). Non-significant results are highlighted by an asterisk, indicating the presence of an effect (i.e., the observed effect is not equivalent to zero).

To sum up, none of our three dependent variables revealed interactions between the factors Entrainment and Phase that were driven by differences between IP and OP in the rhythmic condition, which would have been an indicator of attentional phase entrainment in support of our main hypothesis. Rather, the most prominent pattern was an increase in behavioral performance at the medium and late compared to the early target time points. In addition, only RTs revealed a weak evidence in support of an overall facilitation by rhythmic compared to arhythmic sensory stimulation.

## 4. Discussion

We set out to test the effects of 10 Hz rhythmic tactile entrainment on behavioral performance. Contrary to our main hypothesis, we did not observe phase-specific effects and only weak evidence for overall differences following rhythmic versus arhythmic tactile stimulation.

### 4.1. No evidence for phase entrainment

If rhythmic but not arhythmic tactile stimulation would have caused subsequent rhythmic fluctuations in the processing of tactile targets, we would have expected an interaction between the factors Entrainment and Phase, with larger performance differences between in-phase compared to out-of-phase targets in the rhythmic versus the arhythmic condition. However, only RT data showed such an interaction, and surprisingly in the opposite direction, with faster responses to out-of-phase compared to in-phase targets only in the arhythmic condition (Figure 2B). Crucially, this indicates that the present protocol was not able to induce behaviorally relevant phase-entrainment in the tactile modality.

There are a number of potential reasons for this finding. On the one hand, it is possible that behaviorally relevant sensory entrainment is generally not possible in the tactile domain. As reviewed above, studies so far have either induced behaviorally relevant entrainment via direct cortical electrical or magnetic stimulation (Ruzzoli and Soto-Faraco, 2014; Gundlach et al., 2016), or did not report behavioral results (Fabbrini et al., 2022; Houlgreave et al., 2022). In line with such an interpretation, several recent studies have failed to demonstrate entrainment also in the visual and auditory domain, thus questioning the ubiquity of the phenomenon and suggesting that entrainment might depend on very specific task and stimulus parameters (de Graaf and Duecker, 2021; Lin et al., 2021; Pomper et al., 2022).

On the other hand, it is also possible that the occurrence of (somatosensory) entrainment requires very specific stimulus parameters. First, we presently only tested entrainment with a fixed frequency of 10 Hz. While previous studies used rhythms in the same range, at least some of them adjusted the specific frequency to the participants’ individual alpha-band (Gundlach et al., 2016; Fabbrini et al., 2022; but see Ruzzoli and Soto-Faraco, 2014). However, such an individual frequency specificity of entrainment would render the phenomenon practically irrelevant for everyday life outside the lab, as external rhythmic stimuli will rarely exactly fit an individual’s ongoing alpha frequency.

Second, it is possible that tactile entrainment is more readily elicited using other typical neural activity frequencies, e.g., in the theta (4–7 Hz) or delta range (1–4 Hz). While tactile entrainment has so far only been investigated in the alpha range, there are reports of successful entrainment in the visual and auditory domain using lower frequencies (Lawrance et al., 2014; Hickok et al., 2015; Barnhart et al., 2018; Bauer et al., 2021), which should be further investigated in future studies.

Third, regardless of the specific stimulation rate, individuals might show differences in the phase and exact frequency of entrainment effects (Henry and Obleser, 2012; Landau and Fries, 2012). Testing subsequent target processing at only three in-phase and out-of-phase time points might not be sensitive enough to account for such variability. Future studies could employ behavioral dense sampling approaches (Landau and Fries, 2012; Pomper and Ansorge, 2021) to test attentional modulations at a much finer timescale.

It is worth noting, however, that substantial individual differences in the frequency and/ or phase of the entrainment effect (i.e., the brain’s entrainment ‘response’ to a given regular stimulation, not the optimal frequency of that stimulation itself) seem to contradict the presumed function of entrainment. If the mechanism of entrainment serves to align ongoing fluctuations in neural excitability with a regular external stimulus in order to facilitate the processing of that stimulus in the future, then the frequency and phase of that entrainment effect should be highly similar to that of the stimulus, and as a consequence, also highly similar between subjects.

Finally, entrainment in the somatosensory modality might be more restricted to the stimulation period and not extending beyond in time. Whether this is the case would be an interesting goal for a follow up experiment. However, the present study was designed closely to previous reports of entrainment in the visual and auditory domain, which demonstrated effects outlasting the stimulation period (e.g., Mathewson et al., 2012; de Graaf et al., 2013; Spaak et al., 2014; Hickok et al., 2015). As such, it was our primary present goal to establish and test a similar experimental protocol in the tactile domain.

In summary, parameters such as the frequency of the entrainment stimulus, interindividual differences in phase and frequency of the subsequent attentional fluctuations, the temporal extend of entrainment might differ between sensory modalities, and should be further investigated within the somatosensory domain.

### 4.2. Effects of foreperiod and expectation

Why did the current protocol produce differences in RTs between in-phase and out-of-phase targets in the arhythmic condition (Figure 2B), in which the entrainment phase did not convey any meaningful phase information? Compared to the rhythmic condition, the end of the entrainment and the beginning of the target interval are probably more difficult to anticipate and prepare for in the arhythmic condition, whose stimuli offer little temporal information. This likely caused slower RTs for targets presented at the early time-window, and even more so for the in-phase targets, which appeared 50 ms prior to the out-of phase targets (see Figure 2A). Additionally, faster RTs following rhythmic compared to arhythmic entrainment in the early time-window might also be attributed to higher saliency of regular compared to irregular sequences (Zhao et al., 2013; Southwell et al., 2017), with the resulting increased attention following rhythmic entrainment facilitating target detection, particularly at the early interval.

More generally, the effect of anticipation and preparation is further reflected in the presently overall lower behavioral performance in the early- compared to the later time-windows. This is in line with a large body of research on the foreperiod effect (Niemi and Näätänen, 1981; Han and Proctor, 2022), demonstrating increasing behavioral performance with the passage of time between a cue or warning stimulus and a target.

### 4.3. Conclusion

Despite using a protocol close to previous successful reports of behavioral phase-entrainment in the visual and auditory domain, we presently were not able to observe a similar effect using tactile stimuli. As several recent entrainment studies likewise reported null findings, future research should comprehensively investigate the necessary stimulus and task parameters leading to this phenomenon.

## Data availability statement

The raw data supporting the conclusions of this article will be made available by the authors, without undue reservation.

## Ethics statement

Ethical review and approval was not required for the study on human participants in accordance with the local legislation and institutional requirements. The patients/participants provided their written informed consent to participate in this study.

## Author contributions

UP designed the study, conducted the research, analyzed the data, and wrote the manuscript.

## Funding

This research was supported by an Austrian Science Fund ‘Young Independent Researcher Groups’ grant (ZK66) to UP.

## Conflict of interest

The author declares that the research was conducted in the absence of any commercial or financial relationships that could be construed as a potential conflict of interest.

## Publisher’s note

All claims expressed in this article are solely those of the authors and do not necessarily represent those of their affiliated organizations, or those of the publisher, the editors and the reviewers. Any product that may be evaluated in this article, or claim that may be made by its manufacturer, is not guaranteed or endorsed by the publisher.

## Footnotes

^1^Note that in the following, we refer to ‚entrainment‘to describe rhythmic sensory stimulation, which in the past has been reported to lead to frequency-specific neural synchronization, despite the fact that presently only behavioural data were collected.
